# A Theoretical Study on an Elastic Polymer Thin Film-Based Capacitive Wind-Pressure Sensor

**DOI:** 10.3390/polym12092133

**Published:** 2020-09-18

**Authors:** Xue Li, Jun-Yi Sun, Bin-Bin Shi, Zhi-Hang Zhao, Xiao-Ting He

**Affiliations:** 1 School of Civil Engineering, Chongqing University, Chongqing 400045, China; 20161602025t@cqu.edu.cn (X.L.); 201916131096@cqu.edu.cn (B.-B.S.); 20135542@cqu.edu.cn (Z.-H.Z.); hexiaoting@cqu.edu.cn (X.-T.H.); 2 Key Laboratory of New Technology for Construction of Cities in Mountain Area (Chongqing University), Ministry of Education, Chongqing 400045, China

**Keywords:** capacitive pressure sensor, parallel plate capacitor, structural wind pressure, polymer thin film, closed-form solution

## Abstract

This study is devoted to the design of an elastic polymer thin film-based capacitive wind-pressure sensor to meet the anticipated use for real-time monitoring of structural wind pressure in civil engineering. This sensor is composed of four basic units: lateral elastic deflection unit of a wind-driven circular polymer thin film, parallel plate capacitor with a movable circular electrode plate, spring-driven return unit of the movable electrode plate, and dielectric materials between electrode plates. The capacitance of the capacitor varies with the parallel move of the movable electrode plate which is first driven by the lateral elastic deflection of the wind-driven film and then is, after the wind pressure is reduced or eliminated, returned quickly by the drive springs. The closed-form solution for the contact problem between the wind-driven thin film and the spring-driven movable electrode plate is presented, and its reliability is proved by the experiment conducted. The numerical examples conducted show that it is workable that by using the numerical calibration based on the presented closed-form solution the proposed sensor is designed into a nonlinear sensor with larger pressure-monitoring range and faster response speed than the linear sensor usually based on experimental calibration.

## 1. Introduction

Wind loads may cause cyclic stress in some slender members or structures, such as light poles or wire poles [1], wind-power towers [2], electric transmission towers [3], lifting equipment [4], long-span bridges [5] and ultrahigh-rise buildings [6]. When the stress cycles accumulate to a certain level, fatigue failures are very likely to occur in these structural members or structures. Therefore, to determine the corresponding accumulating cyclic level of cyclic stress at each stress level, the real-time monitoring of the cyclic stress in these structures or structural members is necessary. The structural safety, accumulative damage, and residual fatigue life can thus be scientifically estimated with real-time data monitoring. The wind-induced fatigue problem of structures, however, has always depended on the so-called fatigue design method, which is based on predicting the wind field characteristics of structures [7,8]. At present, real-time monitoring and assessment are rarely used in both mechanical and civil engineering structures. An actual ambient wind field is in fact very complicated and difficult to describe due to the fact that the distribution of wind field is random and discrete, therefore it is usually difficult to predict and grasp the wind field characteristics of structures, especially for ultra-high-rise buildings [9,10]. However, if we monitor the time-dependent acting force of wind on the exterior facade of ultra-high-rise buildings (i.e., the time-dependent wind loads of structure), then based on the relationship between wind loads and wind-induced response of structure which can be determined during structural design, the wind-induced fatigue of structures can be assessed by analyzing the characteristics of wind loads varying with time, such as the maximum value, minimum value, and cyclic frequency of the wind loads. Obviously, such a goal depends almost entirely on a reliable and effective wind-pressure sensor, which is suitable for use in ultra-high-rise buildings, and in particular the cost of this type of sensor should be as low as possible due to the need for a large number of arrangements along the exterior facade of ultra-high-rise buildings. So far, however, there are no wind-pressure sensors that can be used directly in ultra-high-rise buildings.

The monitoring methods in structural health monitoring mainly involve the measurement of force, displacement and strain [11,12,13], in which the method of measuring force is rarely used due to the usually strong force in structural members. The measurement of stress is usually achieved by first measuring strain then transforming the measured strain into stress, rather than by measuring the force in structural members. However, it is usually difficult to ensure that the strain monitoring results, measured by fixing strain gauges [14] or fiber Bragg grating strain measuring instruments [15] onto the structural members of the large-scale structures such as bridges [16,17] and ultrahigh-rise buildings [18,19], can reflect the true strain level of the measured structural member. Usually, the effectiveness of strain monitoring results depends not only on the accuracy of the monitoring instrument itself but also on many factors such as the installation position and construction quality of sensors. These methods for measuring strain could be suitable for some structural components with smaller size, but is usually not suitable for the ones with larger size, especially for the reinforced concrete members with larger size. However, if we directly measure the wind pressure acting on the exterior facade of ultrahigh-rise buildings, we can avoid these strain-monitoring-related problems or difficulties. In addition, the results of wind-pressure monitoring can directly reflect the actual wind-pressure level (i.e., the real external action level of structures), which only depends on the accuracy of the wind-pressure sensors. The existing methods measuring the wind pressure on the surface of structural components or structures can be a direct method, such as pressure valve method [20,21], pressure sensitive coating method [22,23] and pressure sensor method [24,25,26,27], or an indirect method, which is basically measuring wind speed and then converts to wind pressure in some way [28]. The indirect method is often affected by environmental factors such as air humidity and temperature, therefore the direct measuring methods could be regarded as a relatively more precise method in comparison with the direct measuring method. Among these existing direct measuring methods, most of them have some common shortcomings, such as complicated manufacturing process, high production cost, vulnerability to ambient factors, narrow measuring range, so that these methods are not suitable for the use of the long-term real-time monitoring of large-scale structures, especially for the ultrahigh-rise buildings. For an instance, Rossetti et al. [24] developed a capacitive wind-pressure sensor with a ±250 Pa pressure measuring range for wireless wind sail monitoring, where the conductive polymeric films were used to act as the elastic deformation element and the movable electrode plate of the sensing capacitor. The once-in-50-years basic wind pressure in civil engineering is usually in the range of 300 Pa to 1850 Pa, therefore this sensor does not meet the anticipated use for real-time monitoring of structural wind pressure in civil engineering. Another example is An et al. [25] developed a soft capacitive wind-pressure sensor, which employed the soft silicone rubber as the dielectric elastomer between the two electrode plates in the capacitor. In fact, this type of techniques, which is to compress the dielectric elastomer to change the capacitance of the capacitor, has been used in various engineering applications [29,30]. However, the range of elastic deformation of the dielectric elastomer is usually limited, i.e., the ability of recoverable compression deformation of the dielectric elastomer is limited, which means that the pressure measuring range is also limited. The flexible characteristic of the dielectric elastomer should be beneficial for lowering the minimum distinguishable pressure and be also necessary for some soft applications [31]. However, it is also the main reason for viscoelasticity of materials, which may bring the undesirable sensor behaviors [24]. In addition, the long-term real-time monitoring of wind pressure on the exterior facade of ultrahigh-rise buildings requires that the capacitive wind-pressure sensors must be able to adapt to large changes in temperature and humidity.

The existing capacitive pressure sensors always combine the elastic deformation element with the capacitor, i.e., the elastic deformation element is either one of the electrode plates or the dielectric material between the electrode plates, as done in the capacitive pressure sensors using soft dielectric elastomer or conductive thin elastic films [32,33]. This study plans to separate the elastic deformation element form the capacitor. The developed elastic polymer thin film-based capacitive wind-pressure sensor is mainly composed of four basic units: lateral elastic deflection unit of a wind-driven circular polymer thin film, parallel plate capacitor with a movable circular electrode plate, spring-driven return unit of the movable electrode plate, and dielectric materials between electrode plates. The capacitance of the capacitor varies with the parallel move of the movable electrode plate which is first driven by the lateral elastic deflection of the wind-driven film and then is, after the wind pressure is reduced or eliminated, returned quickly by the drive springs. The advantages with this design are mainly reflected in the following aspects: (1) For the elastic deformation element, we can freely select the polymer thin film with excellent elasticity in a wide range of materials and decide its thickness according to the minimum distinguishable pressure of the sensor, which could be much more “free” than finding the films with excellent conductivity and excellent elasticity; (2) Under wind pressure the variation range of the maximum lateral elastic deflection of the circular thin film is far greater than its elastic compression range along its thickness direction, which implicates a greater variation range of capacitance of the parallel plate capacitor; (3) The movable electrode plate and elastic polymer thin film can be quickly returned by springs, reducing the viscoelastic phenomenon in soft pressure sensors; (4) The choice of the dielectric material can only involve the softness and high dielectric coefficient of the material, rather than the resilience of the elastic deformation of the material simultaneously; (5) Parallel plate capacitors and polymer thin films with excellent elasticity have unique feature of low cost manufacturing and are easy to assemble as a whole.

In this paper, the closed-form solution for the contact problem between the wind-driven circular polymer thin film and the spring-driven movable electrode plate is given, and is used to determine the analytical relationship between the wind pressure and the displacement of the movable electrode plate, as well as the analytical relationship between the wind pressure and the capacitance of the capacitor. Therefore, with this closed-form solution the elastic polymer thin film-based capacitive wind-pressure sensor can be designed into a nonlinear sensor with larger pressure measuring range and faster response speed than the linear sensor usually based on experimental calibration. In the following section, the structure and operating principle of the sensor are introduced in detail. In Section 3, the contact problem between the peripherally fixed wind-driven circular polymer elastic thin film and the spring-driven movable electrode plate that is the problem of axisymmetric deformation of the peripherally fixed circular membrane with limited maximum deflection, is analytically solved, and its closed-form solution for deflection and stress is presented. In Section 4, the reliability of the presented closed-form solution is verified by comparing with the well-known Hencky solution and by the experiment conducted. In Section 5, the design of the elastic polymer thin film-based capacitive wind-pressure sensor is clearly illustrated by two examples, including the mathematical modeling and numerical calibration of the sensor. Concluding remarks are presented in Section 6.

## 2. Structure and Operating Principle of the Sensor

**Figure 1:**
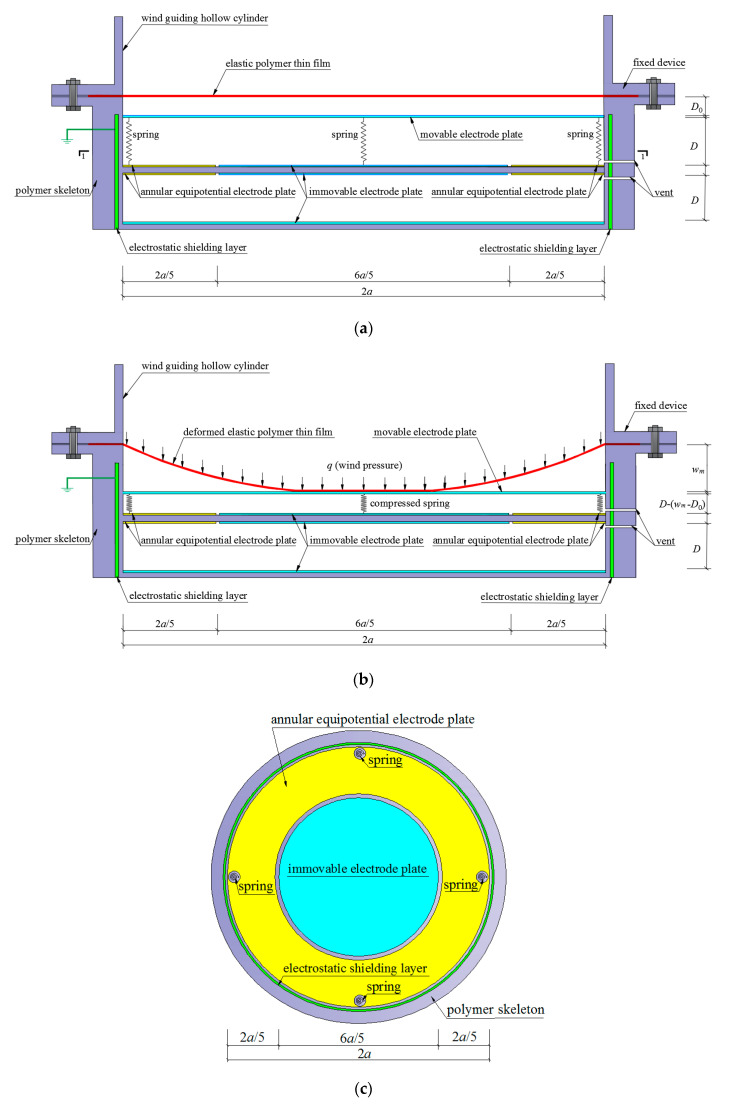
A schematic view of structure and operating principle of the developed capacitive wind-pressure sensor: (**a**) initial state; (**b**) working state; (**c**) sectional view.

The structure and operating principle of the proposed capacitive wind-pressure sensor will be illustrated in this section. The overall configuration of the proposed capacitive wind-pressure sensor includes upper, middle, and lower three parts, as shown in Figure 1.

The upper part is a wind pressure receiving unit of a wind-driven circular polymer thin film with radius *a*, providing a displacement driving force from the lateral elastic deflection *w_m_* of the thin film. For the purpose that the received wind pressure could, as uniformly and transversely as possible, act on the thin film, the wind guiding hollow cylinder is set up at the front end of the proposed sensor. The effectiveness of setting up the wind guiding hollow cylinder will be experimentally tested in Section 4. The middle and lower two parts are two air-spaced circular parallel plate capacitors with the same radius, and each one of the two parts uses an immovable annular equipotential protection electrode plate to eliminate the influence of additional edge capacitance. The leftover part surrounded by the immovable annular equipotential protection electrode plate on the same plane is used as the working electrode plate of the sensor, and its radius is set up to be 3*a*/5. The immovable circular working electrode plate of radius 3*a*/5 and its corresponding immovable annular equipotential protection electrode plate are, on the same plane, insulated electrically from each other, and the distance between them should be as small as possible but should be greater than the distance of dielectric breakdown. As a result, the width of each immovable annular equipotential protection electrode plate is close to but less than 2*a*/5, and the original distance *D* between the upper and lower electrode plates of each parallel plate capacitor should be about *a*/5, in order to produce a better effect of eliminating additional edge capacitance. All electrode plates should be as thin as possible, for example, they can be made of silver or gold coating films of about 0.2 mm, to eliminate the influence of additional edge capacitance as much as possible.

The circular parallel plate variable capacitor in the middle part is used as a working capacitor, and its capacitance will vary along with the movement of a movable circular electrode plate that can be formed from a very light rigid plate coated by silver or gold. The circular parallel plate fixed capacitor in lower part is used as only a reference capacitor to eliminate the influence of the change of dielectric constant caused by the change of air humidity. As is known to all, the air dielectric constant is very easy to be affected by the change of air humidity, while the capacitive wind-pressure sensors here proposed are developed mainly for the application on the exterior facade of ultrahigh-rise buildings. Therefore, the change of air humidity is inevitable, then the change of dielectric constant cannot also be avoided, but the ratio of the capacitance measured values of the variable and fixed capacitors has nothing to do with the air dielectric constant. This is why the reference capacitor is set up, which will be seen from the derivation from Equations (1) through (3).

The distance *D_0_* between the initially flat thin film and the initial position of the movable circular electrode plate is set up for adjusting the minimum distinguishable wind pressure of the sensor, which may be seen from Figure 1a and the derivation from Equations (51) through (54). The lateral elastic deflection of the wind-driven circular polymer thin film, when greater than the distance *D_0_*, pushes the movable circular electrode plate to move in parallel, resulting in the capacitance changes of the circular parallel plate variable capacitor. The springs are used to drive the movable circular electrode plate to return quickly to its initial position, after the wind pressure is reduced or eliminated. An initial compression length △*l* of springs is set up for adjusting the return speed of the movable circular electrode plate. Therefore, the capacitive wind-pressure sensor developed here does not give rise to the viscoelastic phenomenon which is very easy to be present in soft pressure sensors.

The electrical capacitance of the circular parallel plate variable capacitor is given by *C_0_* when the movable circular electrode plate is in its initial position as shown in Figure 1a, i.e.,

(1) $$C_{0}=\frac{\varepsilon_{0}\varepsilon_{r}\pi {\left(3a/5\right)}^{2}}{D},$$

and by *C* when the movable circular electrode plate left its initial position as shown in Figure 1b, i.e.,

(2) $$C=\frac{\varepsilon_{0}\varepsilon_{r}\pi {\left(3a/5\right)}^{2}}{D+D_{0}-w_{m}},$$

where *ε*_0_ describes the vacuum permittivity and *ε_r_* is the relative permittivity of air. Meanwhile, the electrical capacitance of the circular parallel plate fixed capacitor always keeps *C_0_* constant. Therefore, if we simultaneously measure the electrical capacitance of both variable capacitor and fixed capacitor, then it is possible to combine Equations (1) and (2) into the following relationship

(3) $$\frac{C_{0}}{C}=\frac{D+D_{0}-w_{m}}{D}.$$

The influence of the change of air humidity on the value of *ε_r_* is thus eliminated.

From Equation (3) and Figure 1b we may realize that if the wind pressure *q* can be expressed into a function of the maximum lateral elastic deflection *w_m_* of the thin film, then it can further be expressed into a function of *C_0_*/*C* by Equation (3). To this end, in the following section we will analytically solve the contact problem between the peripherally fixed wind-driven circular polymer elastic thin film and the spring-driven movable electrode plate, as shown in Figure 1b, which can, in mechanics, be simplified as a problem of axisymmetric deformation of a peripherally fixed circular membrane with elastically restricted maximum deflection under the action of uniformly distributed transverse loads *q*.

## 3. Analytical Solution to the Mechanical Model

**Figure 2:**
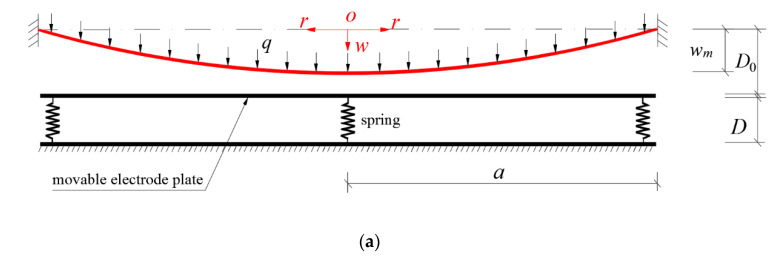

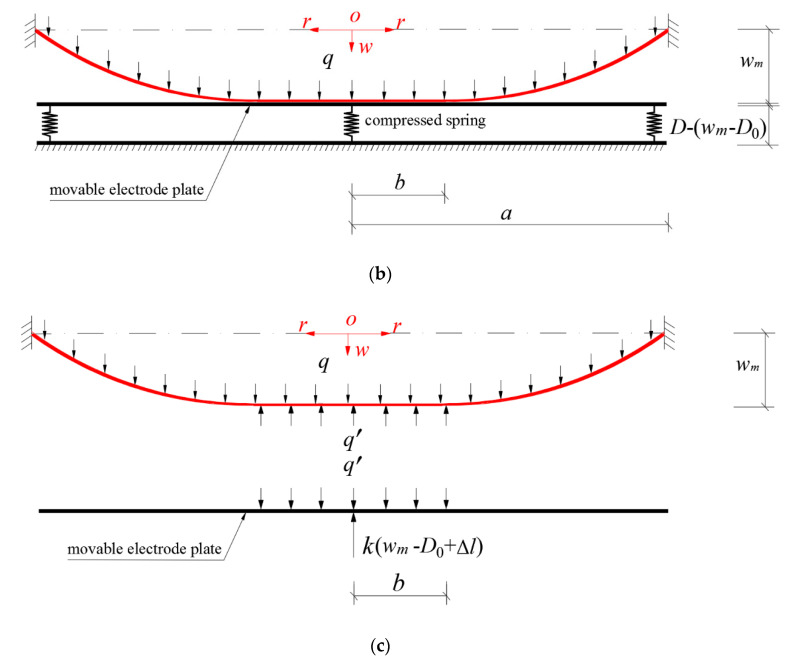
Sketch of the contact problem between wind-driven circular membrane and spring-driven frictionless rigid plate: (**a**) non-contact state between membrane and plate; (**b**) contact state between membrane and plate; (**c**) anatomical view of interaction force between deflected membrane and spring-driven plate.

The problem before the peripherally fixed wind-driven circular polymer elastic thin film touches the spring-driven movable electrode plate is simplified into the well-known Föppl–Hencky membrane problem, i.e., the problem of axisymmetric deformation of a peripherally fixed circular membrane under the action of uniformly distributed transverse loads *q* [34,35,36,37,38,39,40], as shown in Figure 2a. The effectiveness of the well-known Hencky solution is recognized. Figure 2b represents the problem after the peripherally fixed wind-driven circular polymer elastic thin film touches the spring-driven movable electrode plate, i.e., the problem of axisymmetric deformation of a peripherally fixed circular membrane with elastically restricted maximum deflection under the action of uniformly distributed transverse loads *q*, which will be dealt with below. The difference between the problem dealt herein and the problem previously dealt in [41,42] is solely that one is elastic restriction on maximum deflection while the other one is fixed restriction. However, such a small difference in physics will give rise to serious analytical difficulties in mathematics, which will be seen below.

Suppose that an initially flat peripherally fixed circular membrane with Young’s modulus *E*, Poisson’s ratio *v*, thickness *h* and radius *a* is subjected to a uniformly distributed transverse loads *q*, as shown in Figure 2a, and as the loads *q* intensify, it will then come into contact with a smooth frictionless rigid plate (i.e., the movable electrode plate in Figure 1) which is always under the action of the springs with stiffness coefficient *k* and initial compressed length Δ*l*, as shown in Figure 2b. In Figure 2, the dash-dotted line represents the geometric middle plane of the initially flat circular membrane, in which the polar coordinates plane (r,φ) of the cylindrical coordinates system (r,φ,w) locates, where *r*, φ and *w* represent the radial, circumferential, and transverse coordinates, *b* represents the contact radius between the deflected circular membrane and the spring-driven frictionless movable rigid electrode plate, *D_0_* is the initial distance between the initially flat circular membrane and the initial position of the frictionless movable rigid electrode plate, *D* represents the initial distance between the movable and immovable electrode plates in Figure 1, *q^′^* represents the interaction force between the deflected circular membrane and the spring-driven frictionless movable rigid electrode plate, *w_m_* represents the maximum deflection of the circular membrane and $w_{m}=w(b)$ for the contact state between the deflected membrane and the spring-driven frictionless rigid plate.

**Figure 3:**
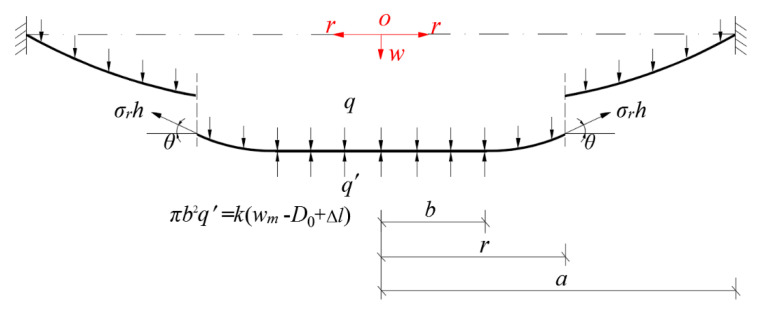
The free body diagram of the membrane within b≤r≤a.

Let us take a piece of the central portion of the whole deformed circular membrane whose radius is b≤r≤a, to study the static problem of equilibrium of this piece of the deformed circular membrane under the joint actions of the transverse loads q, reaction force $q^{\prime }$ from the spring-driven rigid plate, and the membrane force $\sigma_{r}h$ acting on the boundary of radius r, as shown in Figure 3, where $\sigma_{r}$ is the radial stress and θ is the meridional rotation angle of the deflecting membrane.

In the vertical direction perpendicular to the initially flat circular membrane, there are three vertical forces, i.e., the applied force $\pi r^{2}q$, reaction force $\pi b^{2}q^{\prime }$ and the vertical membrane force $2\pi r\sigma_{r}h\sin\theta$. Therefore, the so-called out-of-plane equilibrium equation is

(4) $$2\pi r\sigma_{r}h\sin\theta =\pi r^{2}q-\pi b^{2}q^{\prime },$$

where $\pi b^{2}q^{\prime }=k(w_{m}-D_{0}+\Delta l)$. Since θ=0 at r=b, i.e., sinθ=0 at r=b, from Equation (4) it is easy to be found that

(5) $$q^{\prime }=q.$$

Substituting Equation (5) into Equation (4) yields

(6) $$2\pi r\sigma_{r}h\sin\theta =\pi (r^{2}-b^{2})q,$$

where

(7) $$\sin\theta =-\frac{dw}{dr}.$$

Substituting Equation (7) into Equation (6), one has

(8) $$(r^{2}-b^{2})q+2r\sigma_{r}h\frac{dw}{dr}=0.$$

In the horizontal direction parallel to the initially flat circular membrane, there are the joint actions of the circumferential membrane force $\sigma_{t}h$ and the horizontal component of the radial membrane force $\sigma_{r}h$, where $\sigma_{t}$ denotes circumferential stress. The so-called in-plane equilibrium equation may be written as

(9) $$\frac{d}{dr}(r\sigma_{r})-\sigma_{t}=0.$$

The relations of the strain and displacement of the large deflection problem may be written as

(10) $$e_{r}=\frac{du}{dr}+\frac{1}{2}{\left(\frac{dw}{dr}\right)}^{2}$$

and

(11) $$e_{t}=\frac{u}{r}.$$

where $e_{r}$, $e_{t}$ and u denote the radial strain, circumferential strain, and the radial displacement, respectively. The relations of the stress and strain are

(12) $$\sigma_{r}=\frac{E}{1-\nu^{2}}(e_{r}+\nu e_{t})$$

and

(13) $$\sigma_{t}=\frac{E}{1-\nu^{2}}(e_{t}+\nu e_{r}).$$

Substituting Equations (10) and (11) into Equations (12) and (13) yields

(14) $$\sigma_{r}=\frac{E}{1-\nu^{2}}[\frac{du}{dr}+\frac{1}{2}{\left(\frac{dw}{dr}\right)}^{2}+\nu \frac{u}{r}]$$

and

(15) $$\sigma_{t}=\frac{E}{1-\nu^{2}}[\frac{u}{r}+\nu \frac{du}{dr}+\frac{\nu }{2}{\left(\frac{dw}{dr}\right)}^{2}].$$

By means of Equations (14), (15) and (9), one has

(16) $$\frac{u}{r}=\frac{1}{E}(\sigma_{t}-\nu \sigma_{r})=\frac{1}{E}[\frac{d}{dr}(r\sigma_{r})-\nu \sigma_{r}].$$

Substituting the u of Equation (16) into Equation (14), it is found that

(17) $$r\frac{d}{dr}[\frac{1}{r}\frac{d}{dr}(r^{2}\sigma_{r})]+\frac{E}{2}{\left(\frac{dw}{dr}\right)}^{2}=0.$$

The detailed derivation from Equation (9) to Equation (17) can be found from any general theory of plates and shells [43], so it is not necessary to discuss it here. In addition, from the above derivation, it is not difficult to find that Equations (8) and (17) are two equations for the solutions of $\sigma_{r}$ and w.

On the other hand, in the central contact portion (0<r≤b) between the deformed circular membrane and the spring-driven frictionless rigid plate, due to dw/dr=0, Equations (10) and (11) become

(18) $$e_{r}=\frac{du}{dr}$$

and

(19) $$e_{t}=\frac{u}{r}.$$

Substituting Equations (18) and (19) into Equations (12) and (13) yields

(20) $$\sigma_{r}=\frac{E}{1-\nu^{2}}(\frac{du}{dr}+\nu \frac{u}{r})$$

and

(21) $$\sigma_{t}=\frac{E}{1-\nu^{2}}(\frac{u}{r}+\nu \frac{du}{dr}).$$

Substituting Equations (20) and (21) into Equation (9) yields

(22) $$r\frac{d^{2}u}{dr^{2}}+\frac{du}{dr}-\frac{u}{r}=0.$$

Obviously, Equation (22) satisfies the form of the Euler equation, and its general solution may be written as

(23) $$u(r)=C_{1}r+C_{2}\frac{1}{r}.$$

where $C_{1}$ and $C_{2}$ are two undetermined constants. The conditions to determine $C_{1}$ and $C_{2}$, or the conditions that the special solution of Equation (22) must satisfy, are u=0 at r=0 and u=u(b) at r=b. Therefore, with this boundary conditions it is found that $C_{1}=u(b)/b$ and *C*_2_ ≡ 0. Hence, the special solution of Equation (22) may be written as

(24) $$u(r)=\frac{u(b)}{b}r.$$

Substituting Equation (24) into Equations (18)–(21), it is found that

(25) $$e_{r}=e_{t}=\frac{u(b)}{b}\;\mathrm{in}\;0<r\leq b$$

and

(26) $$\sigma_{r}=\sigma_{t}=\frac{E}{1-\nu }\frac{u(b)}{b}\;\mathrm{in}\;0<r\leq b.$$

Now let us continue addressing the contact problem here. Based on the above derivations, the boundary conditions and continuous conditions for the contact problem may be written as

(27) w=0 at r=a,

(28) $$e_{t}=\frac{u}{r}=\frac{1}{Eh}(\sigma_{t}h-\nu \sigma_{r}h)=0\;\mathrm{at}\;r=a$$

and

(29) $${\left(e_{t}\right)}_{A}={\left(e_{t}\right)}_{B}=\frac{u(b)}{b}\;\mathrm{at}\;r=b,$$

(30) $${\left(\sigma_{r}\right)}_{A}={\left(\sigma_{r}\right)}_{B}=\frac{E}{1-\nu }\frac{u(b)}{b}\;\mathrm{at}\;r=b,$$

(31) $$k(w_{m}-D_{0}+\Delta l)=\pi b^{2}q\;\mathrm{at}\;r=b,$$

where the subscripts A and B denote the regions on two sides of the inter-connecting circle (r=b). The side of region A is under the plane state of radial tensile or compression of the membrane within 0<r≤b, while the side of region B is under the deflection state of the membrane within b≤r≤a.

Let us proceed to the following nondimensionalization

(32) $$Q=\frac{qa}{Eh},\;W=\frac{w}{a},\;S_{r}=\frac{\sigma_{r}}{E},\;S_{t}=\frac{\sigma_{t}}{E},\;x=\frac{r}{a},\;\alpha =\frac{b}{a},\;K=\frac{k}{\pi Eh},\;L=\frac{\Delta l}{a},D^{\circ }=\frac{D_{0}}{a},$$

and transform Equations (8), (16), (17) and Equation (27) to Equation (31) into

(33) $$(x^{2}-\alpha^{2})Q+2xS_{r}\frac{dW}{dx}=0,$$

(34) $$x^{2}\frac{d^{2}S_{r}}{dx^{2}}+3x\frac{dS_{r}}{dx}+\frac{1}{2}{\left(\frac{dW}{dx}\right)}^{2}=0,$$

(35) $$S_{t}\begin{array}{c} = \end{array}S_{r}+x\frac{dS_{r}}{dx},$$

(36) W=0 at x=1,

(37) $$S_{t}-\nu S_{r}=0\;\mathrm{at}\;x=1$$

and

(38) $${\left(S_{t}-\nu S_{r}\right)}_{A}={\left(S_{t}-\nu S_{r}\right)}_{B}=\frac{u(b)}{b}\;\mathrm{at}\;x=\alpha ,$$

(39) $${\left(S_{r}\right)}_{A}={\left(S_{r}\right)}_{B}=\frac{1}{1-\nu }\frac{u(b)}{b}\;\mathrm{at}\;x=\alpha ,$$

(40) $$K(W_{m}-D^{\circ }+L)=\alpha^{2}Q\;\mathrm{at}\;x=\alpha .$$

Eliminating dW/dx from Equations (33) and (34) yields

(41) $$8x^{4}{S_{r}}^{2}\frac{d^{2}S_{r}}{dx^{2}}+24x^{3}{S_{r}}^{2}\frac{dS_{r}}{dx}+{\left(x^{2}-\alpha^{2}\right)}^{2}Q^{2}=0.$$

Expand $S_{r}$ and W to the power series of the x−(1+α)/2

(42) $$S_{r}=Q^{2/3}\sum_{i=0}^{\infty }c_{i}{\left(x-\frac{1+\alpha }{2}\right)}^{i}$$

and

(43) $$W=Q^{1/3}\sum_{i=0}^{\infty }d_{i}{\left(x-\frac{1+\alpha }{2}\right)}^{i}.$$

After substituting Equation (42) into Equation (41), the coefficients $c_{i}$ (i=1,2,3,4,…) can be expressed into the polynomials with regard to $c_{0}$, $c_{1}$ and α, which are shown in Appendix A, where $c_{0}$, $c_{1}$ and α are three undetermined constants. Further substituting Equations (42) and (43) into Equation (33), the coefficients $d_{i}$ (i=1,2,3,4,…) can also be expressed into the polynomials with regard to $c_{0}$, $c_{1}$ and α, which are shown in Appendix B, while $d_{0}$ is another undetermined constant.

The values of undetermined coefficients $c_{0}$, $c_{1}$, α and $d_{0}$ depend on the concrete problem, and can be determined by using the above boundary conditions and continuous conditions. From Equation (43), Equations (36) and (40) give

(44) $$Q^{1/3}\sum_{i=0}^{\infty }d_{i}{\left(\frac{1-\alpha }{2}\right)}^{i}=0$$

and

(45) $$Q^{1/3}\sum_{i=0}^{\infty }d_{i}{\left(\frac{\alpha -1}{2}\right)}^{i}=\frac{\alpha^{2}Q}{K}-L+D^{\circ }.$$

Then Equation (45) minus Equation (44) yields

(46) $$Q^{1/3}\sum_{i=1}^{\infty }d_{i}[{\left(\frac{\alpha -1}{2}\right)}^{i}-{\left(\frac{1-\alpha }{2}\right)}^{i}]=\frac{\alpha^{2}Q}{K}-L+D^{\circ }.$$

From Equations (35) and (42), Equations (37), (38) and (39) give

(47) $$Q^{2/3}(1-\nu )\sum_{i=0}^{\infty }c_{i}{\left(\frac{1-\alpha }{2}\right)}^{i}+Q^{2/3}\sum_{i=1}^{\infty }ic_{i}{\left(\frac{1-\alpha }{2}\right)}^{i-1}=0,$$

(48) $$Q^{2/3}(1-\nu )\sum_{i=0}^{\infty }c_{i}{\left(\frac{\alpha -1}{2}\right)}^{i}+Q^{2/3}\alpha \sum_{i=1}^{\infty }ic_{i}{\left(\frac{\alpha -1}{2}\right)}^{i-1}=\frac{u(b)}{b}$$

and

(49) $$Q^{2/3}\sum_{i=0}^{\infty }c_{i}{\left(\frac{\alpha -1}{2}\right)}^{i}=\frac{1}{1-\nu }\frac{u(b)}{b}.$$

Eliminating u(b) from Equations (48) and (49) it is found that

(50) $$\sum_{i=1}^{\infty }ic_{i}{\left(\frac{\alpha -1}{2}\right)}^{i-1}=0.$$

Therefore, for the problem in which the values of a, h, E, ν and q are known beforehand, the undetermined constants $c_{0}$, $c_{1}$ and α can be determined by the simultaneous solutions of Equations (46), (47) and (50). Furthermore, substituting the known $c_{0}$, $c_{1}$ and α into Equation (44) or Equation (45), the last undetermined constant $d_{0}$ can also be determined. The problem dealt with here is thus solved.

## 4. Effectiveness of the Closed-Form Solution Presented in Section 3

**Figure 4:**
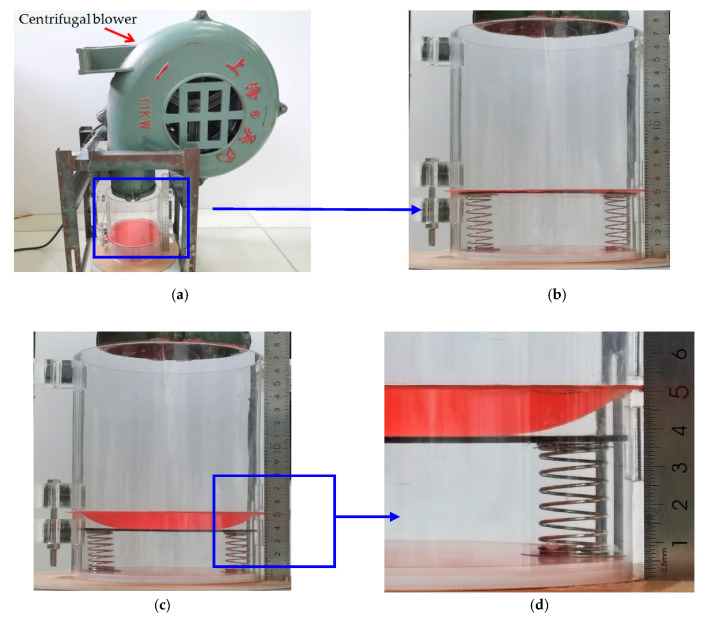

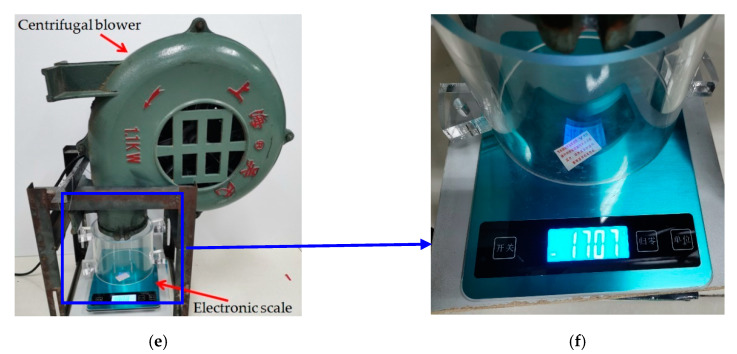
Experimental setup. (**a**–**d**) The case of loading the thin film, where (**a**) full view and (**b**–**d**) enlarged view; (**e**–**f**) The case of loads measurement, where (**e**) full view and (**f**) enlarged view.

To assess the effectiveness of the closed-form solution presented in Section 3, we conducted a simple experiment. The photos of the experimental setup are shown in Figure 4. A piece of thin synthesized latex film (2-ethyl hexyl methacrylate) with elastic modulus $E=3.01\times 10^{6}$ Pa, Poisson’s ratio ν=0.45 and thickness h=0.3 mm is clamped by the two round ends of two transparent Acrylic hollow cylinders with inner radius a=70mm, and the lower surface of the circular polymer thin film is in contact with a movable circular Acrylic plate with radius a=69.5 mm and thickness $t_{1}=2$ mm. The lower surface of the movable circular Acrylic plate is connected to two springs with stiffness coefficient k=2×0.1867 N/mm=0.3734 N/mm, uncompressed original length L=43.05 mm, and initial compressed length $l_{0}=0.05$ mm. A centrifugal blower with AC 220 V and 1.1 KW was used to produce a 1707 × 10^−3^ kg (16.73 N) force, which is acting on the upper surface of the circular polymer thin film with radius 70 mm, converted to the transverse uniformly distributed loads q=1086.80 Pa, the wind pressure value per unit area on the circular plane with radius 70 mm.

**Figure 5:**
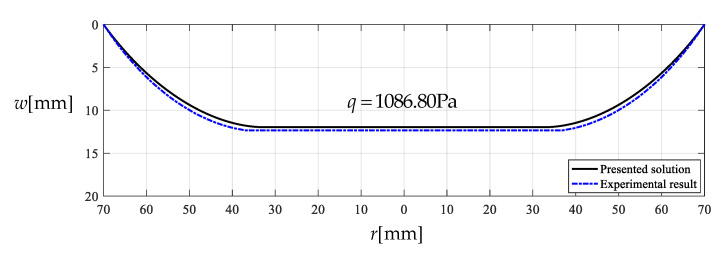
Deflection profiles for *q* = 1086.80 Pa, drawn by experimental data and analytical solution.

Under the action of q=1086.80  Pa, the actually measured value of the maximum deflection of this circular polymer thin film is about $w_{m}=12.35\;\mathrm{mm}$ while the theoretical value calculated by the closed-form solution presented in Section 3 is about $w_{m}=11.97\;\mathrm{mm}$. Figure 5 shows the deflection profiles of this circular polymer thin film, where the solid line represents the results theoretically calculated by the presented closed-form solution and the dash-dotted line represents the experimental results. From Figure 5 it can be seen that the solid line is very close to the dash-dotted line, which indicates that the closed-form solution presented here is basically reliable, from an experimental point of view.

**Figure 6:**
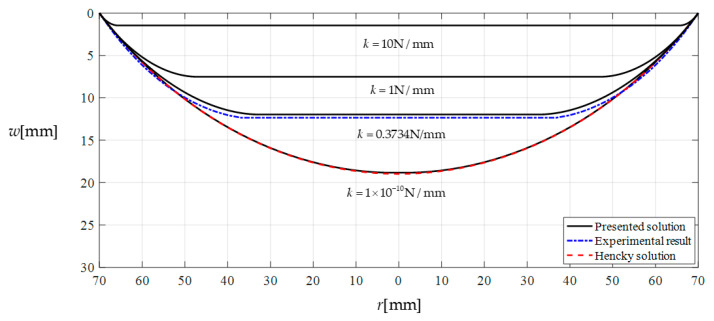
Variations of *w* with *r* when *k* takes 10 N/mm, 1 N/mm, 0.3734 N/mm and 1 × 10^−10^ N/mm, respectively, where *q* keeps 1086.80 Pa.

To further prove the effectiveness of the closed-form solution presented in Section 3, a numerical analysis is conducted as follows. Suppose that we continue adopting all the conditions of this experiment example except for the spring stiffness coefficient *k*, i.e., the elastic modulus $E=3.01\times 10^{6}\;\mathrm{Pa}$, Poisson’s ratio ν=0.45, thickness h=0.3  mm, and radius a=70  mm. The transverse uniformly distributed loads (the wind pressure value per unit area) still takes q=1086.80  Pa; the springs still take uncompressed original length L=43.05  mm and initial compressed length Δl=0.05  mm; while the spring stiffness coefficient *k* takes 10 N/mm, 1 N/mm, 0.3734 N/mm and 1 × 10^−10^ N/mm, respectively. Figure 6 shows the deflection profiles of this circular polymer thin film, where the solid lines represent the results theoretically calculated by the closed-form solution presented in this paper, the dash-dotted line represents the experimental results, and the dashed line represents the results theoretically calculated by the well-known Hencky solution for non-contact issues. It can be seen from Figure 6 that the horizontal segment of the solid lines becomes shorter and shorter as the stiffness coefficient *k* of springs decreases, until *k* takes 1 × 10^−10^ N/mm the horizontal segment becomes invisible to the naked eye. This shows that the contact area between the circular polymer thin film and the movable circular electrode plate of the parallel plate capacitor becomes smaller and smaller as the stiffness coefficient *k* of springs decreases, until *k* takes 1 × 10^−10^ N/mm the contact area almost vanishes, i.e., when *k* takes 1 × 10^−10^ N/mm the contact problem here almost becomes a non-contact issue. From Figure 6 it can be clearly seen that the solid line drawn by the closed-form solution obtained in Section 3 are very close to the dashed line drawn by the well-known Hencky solution for non-contact issues. This indicates that the closed-form solution presented here is reliable, as far as the recognized effectiveness of the well-known Hencky solution is concerned.

**Figure 7:**
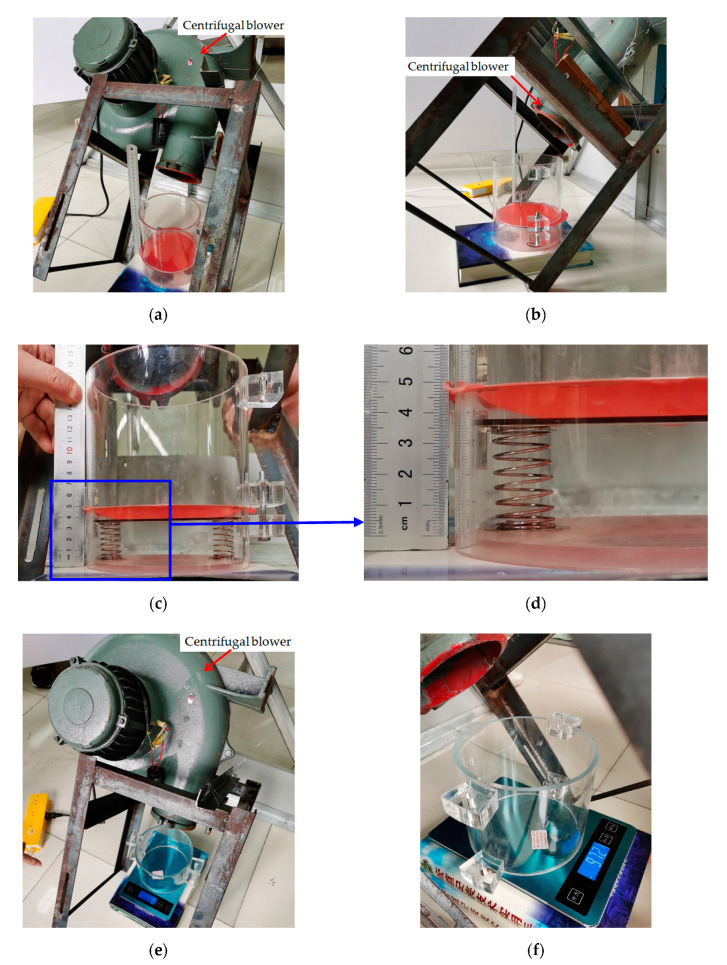
Experimental loading under inclining the centrifugal blower about 45 degrees. (**a**–**d**) The case of loading the thin film, where (**a**) full view and (**b**–**d**) enlarged view; (**e**–**f**) The case of loads measurement, where (**e**) full view and (**f**) enlarged view.

**Figure 8:**
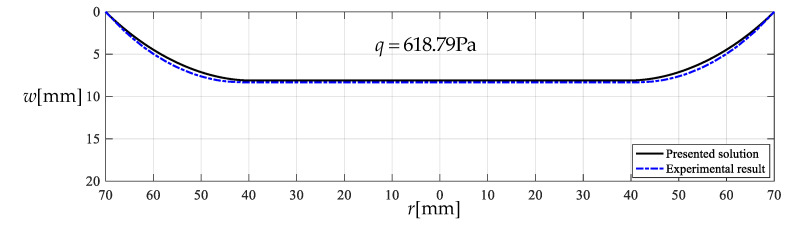
Deflection profiles for *q* = 618.79 Pa, drawn by experimental data and analytical solution.

As is known to all, the direction of winds in nature is random. However, the closed-form solution presented in Section 3 is observed actually to be for uniform transverse loading. Therefore, at the front end of the proposed sensor we set up a wind guiding hollow cylinder such that the received wind pressure could, as uniformly and transversely as possible, act on the thin film, regardless of the direction of winds in nature. Now let us incline the centrifugal blower about 45 degrees, as shown in Figure 7, to test the effectiveness of setting up the wind guiding hollow cylinder. The force produced by the centrifugal blower after inclined 45 degrees is about 972 × 10^−3^ kg (9.53 N), which is converted to the transverse uniformly distributed loads q=618.79  Pa, the wind pressure value per unit area on the circular plane with radius 70 mm. The actually measured value of the maximum deflection of the circular polymer thin film under q=618.79  Pa is about $w_{m}=8.34\mathrm{mm}$, while the theoretical value calculated by the closed-form solution presented in Section 3 is about $w_{m}=8.12\;\mathrm{mm}$. The relative error is about 2.64%. Figure 8 shows the deflection profiles of the deformed thin film, where the solid line represents the results calculated by the closed-form solution presented in Section 3 and the dash-dotted line represents the experimental results. From Figure 8 it can be seen that the solid line is very close to the dash-dotted line, which indicates that the effect of setting up the wind guiding hollow cylinder is basically satisfactory.

## 5. Numerical Calibration of the Sensor

Though the closed-form solution obtained in Section 3 has, in Section 4, been demonstrated to have satisfactory computational accuracy, it can still not be directly used to design the proposed elastic polymer thin film-based capacitive wind-pressure sensor. The main reason herein is that by using the closed-form solution obtained in Section 3, we can only obtain a set of precise numerical calculation values of the maximum deflection *w_m_* and its corresponding loads *q*, rather than the analytical relationship between the loads *q* and the maximum deflection *w_m_*. In other words, the explicit function $q=f(w_{m})$, which is essential to design the sensor proposed here, can still not be derived from the closed-form solution presented in Section 3, because the undetermined constants $c_{0}$, $c_{1}$, α and $d_{0}$ can be determined only under the condition that the loads *q* are known beforehand.

However, the expected explicit function $q=f(w_{m})$ can be obtained by the mathematical modeling based on the precise numerical values of the maximum deflection *w_m_* and its corresponding loads *q*, while these precise numerical values can be calculated by using the closed-form solution obtained in Section 3. The basic modeling thinking is that since the applied total external forces π*a^2^q* is always shared by the deflected circular membrane and the compressed springs, then we can make the contact radius *b* as small as possible by adjusting the initial distance *D_0_* between the initially flat circular membrane and the initial position of the frictionless movable rigid electrode plate, such that the deflected circular membrane in contact problem can roughly be equivalent to a deflected Föppl–Hencky membrane (non-contact problem). The explicit function $q=f(w_{m})$ for a deflected Föppl–Hencky membrane can be derived from the well-known Hencky solution (from Equation (33) in our previous work [35])

(51) $$q=\frac{2Eh}{a^{4}cg^{3}(c)}w_{m}^{3},$$

where *g*( ) is a function and *c* is an undetermined constant which depends, in the well-known Föppl–Hencky membrane problem, on only the value of Poisson’s ratio *v* [35]. Therefore, the mathematical model for the contact problem here can be approximated by

(52) $$\pi a^{2}q=\pi a^{2}\frac{2Eh}{a^{4}cg^{3}(c)}w_{m}^{3}+k(w_{m}-D_{0}+\Delta l),$$

and is further simplified into

(53) $$q=\frac{2Eh}{a^{4}cg^{3}(c)}w_{m}^{3}+\frac{k}{\pi a^{2}}w_{m}+\frac{k(\Delta l-D_{0})}{\pi a^{2}}.$$

Therefore, if we simultaneously measure the electrical capacitance (*C* and *C_0_*) of both variable capacitor and fixed capacitor, then the analytical relationship between the wind pressure *q* and the ratio *C_0_*/*C* of the measured electrical capacitance can, from Equations (3) and (53), be written as

(54) $$q=\frac{2Eh}{a^{4}cg^{3}(c)}{\left(D+D_{0}-D\frac{C_{0}}{C}\right)}^{3}+\frac{k}{\pi a^{2}}(D+D_{0}-D\frac{C_{0}}{C})+\frac{k(\Delta l-D_{0})}{\pi a^{2}}.$$

Obviously, the greater the initial distance *D_0_* is, the smaller the contact radius *b* will be, and the smaller the contact radius *b* is, the more accurate the mathematical model of Equations (53) will be, while the greater the initial distance *D_0_* is, the greater the minimum distinguishable wind pressure of the sensor will be. The acceptable minimum distinguishable wind pressure could be greater than 50–100 Pa, as far as the anticipative use of the proposed sensor for ultrahigh-rise buildings application is concerned. By way of illustration we will show the process or steps how to do this mathematical modeling.

Suppose that we continue adopting all the conditions of the experiment example in Section 4, except for the spring stiffness coefficient *k* and the thickness *h* of the thin film, i.e., the elastic modulus $E=3.01\times 10^{6}\;\mathrm{Pa}$, Poisson’s ratio ν=0.45, and radius a=70  mm, but the thickness h=1  mm, the stiffness coefficient of spring k=0.0015 and 0.01  N/mm, and the initial compressed length of the spring Δl=0.05 and 0.5  mm. The initial distance *D* between the movable and immovable electrode plates takes 14 mm, and the initial distance *D_0_* takes 5 mm. Therefore, based on our previous work [35] the undetermined constant in the well-known Föppl–Hencky membrane problem should be c=0.3380417 due to ν=0.45, hence g(c)=1.105391058. The minimum distinguishable wind pressure is, by Equation (51), calculated to be about 68.643 Pa due to $w_{m}=D_{0}=5\;\mathrm{mm}$.

**Figure 9:**
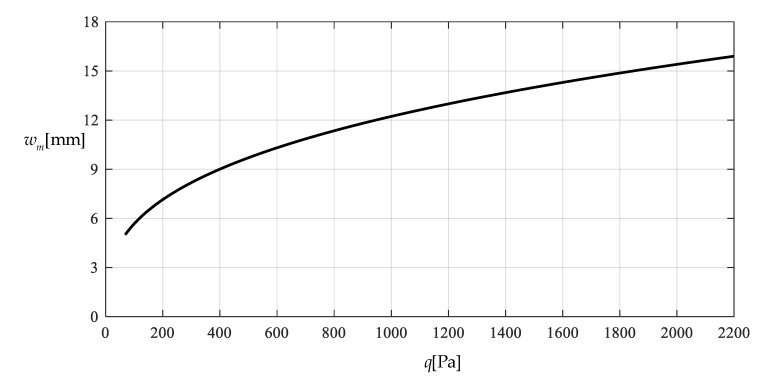
Variations of $w_{m}$ with q.

**Figure 10:**
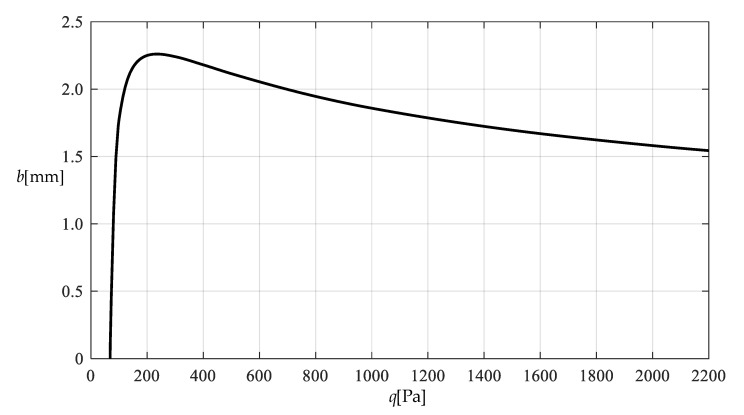
Variation of b with q.

**Figure 11:**
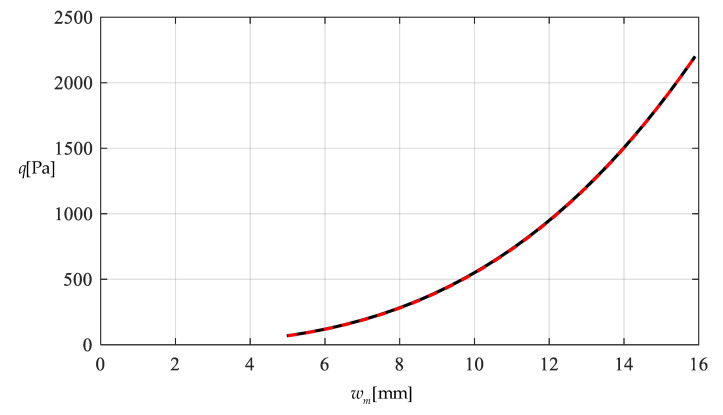
Variations of q with $w_{m}$.

**Figure 12:**
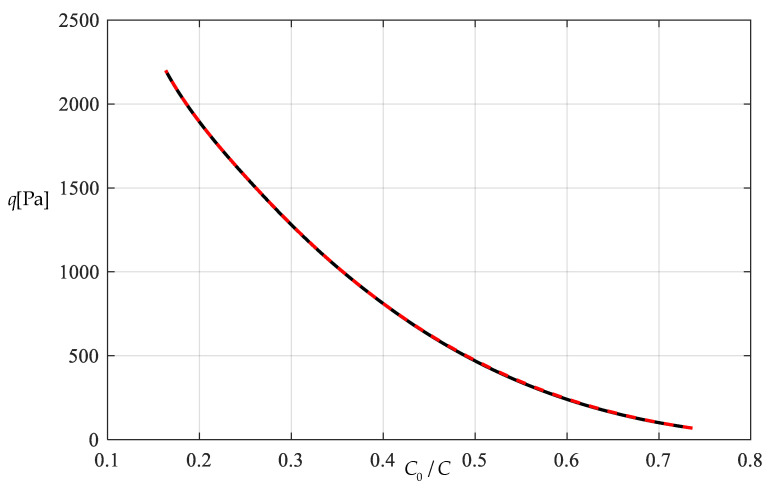
Variations of *q* with *C_0_*/*C*.

Table 1 shows the numerical results for k=0.0015  N/mm and Δl=0.05  mm, in which the maximum deflection *w_m_* and the contact radius *b* are calculated by using the closed-form solution obtained in Section 3 and using the actually applied loads *q*, while the predicted loads $q^{\prime \prime }$ values are calculated by using the prediction model $q^{\prime \prime }=0.549144w_{m}^{3}+0.097442w_{m}-0.482337$ (derived from Equation (53)) and using the calculated *w_m_* values. Figure 9 and Figure 10 show the variations of *w_m_* and *b* with the loads *q*. Figure 11 shows the variations of *q* and $q^{\prime \prime }$ with *w_m_*, where the solid line represents the variation of *q* with *w_m_*, and the dashed line represents the variation of $q^{\prime \prime }$ with *w_m_*. From Figure 11 it can be seen that the dashed line is much closed to the solid line, which means that the computational precision of the prediction model $q^{\prime \prime }=0.549144w_{m}^{3}+0.097442w_{m}-0.482337$ is very well due to the relatively small *b* (see Figure 10). Figure 12 shows the variation of the wind pressure *q* with the ratio *C_0_*/*C* of the measured capacitance, where the dashed line is drawn by the prediction model $q^{\prime \prime }=-1506.851136{\left(C_{0}/C\right)}^{3}+6135.036768{\left(C_{0}/C\right)}^{2}-8327.485513C_{0}/C+3767.947753$, which is derived from Equation (54).

**Figure 13:**
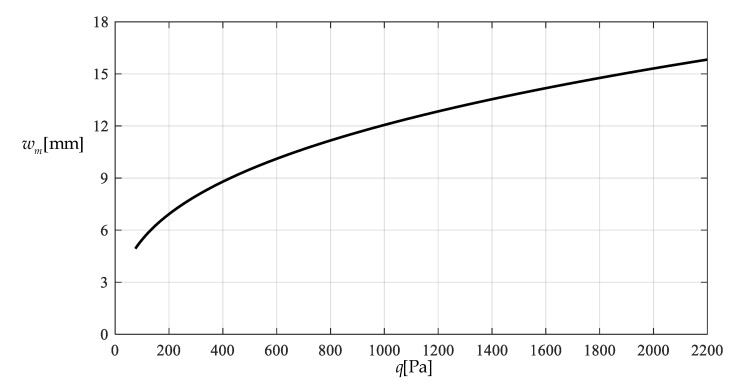
Variation of $w_{m}$ with q.

**Figure 14:**
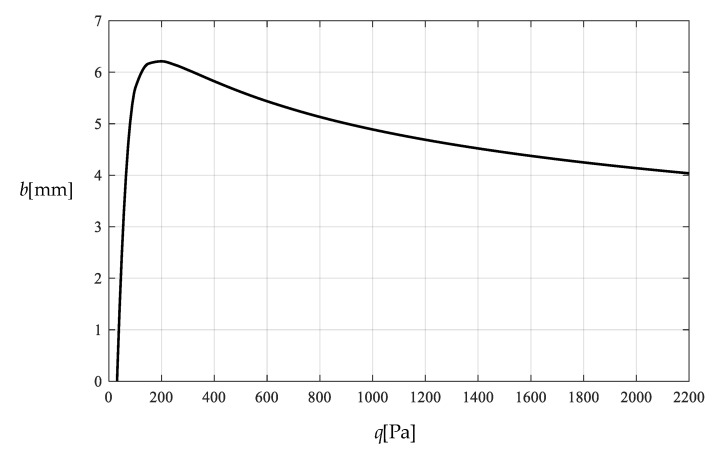
Variation of b with q.

**Figure 15:**
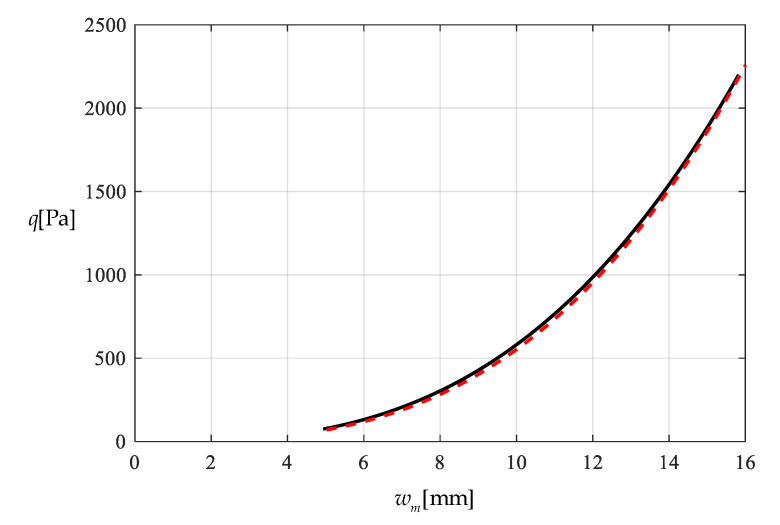
Variations of q with $w_{m}$.

**Figure 16:**
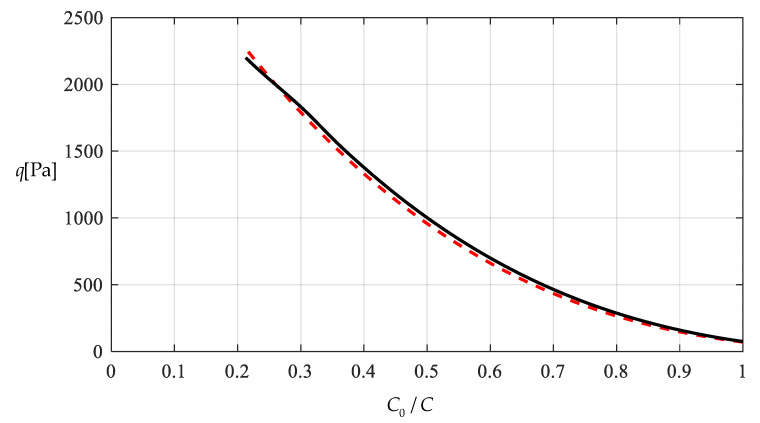
Variations of *q* with *C_0_*/*C*.

Table 2 shows the numerical results for k=0.01  N/mm and Δl=0.5  mm, in which the maximum deflection *w_m_* and contact radius *b* are calculated by using the obtained closed-form solution and using the actually applied loads *q*, while the predicted loads $q^{\prime \prime }$ values are calculated by the prediction model $q^{\prime \prime }=0.549144w_{m}^{3}+0.649612w_{m}-2.923254$ (derived from Equation (53)) and using the calculated *w_m_* values. Figure 13 and Figure 14 show the variations of *w_m_* and *b* with the loads *q*. It can be seen from Figure 10 and Figure 14 that the contact radius *b* in Figure 14 gets much bigger than that in Figure 10 due to the spring stiffness coefficient *k* increased from 0.0015 N/mm to 0.01 N/mm and the spring initial compressed length Δl increased from 0.005 mm to 0.5 mm. Figure 15 shows the variations of *q* and $q^{\prime \prime }$ with *w_m_*, where the solid line represents the variation of *q* with *w_m_*, and the dashed line represents the variation of $q^{\prime \prime }$ with *w_m_*. From Figure 15 it can be seen that the solid line and dashed line diverge slightly in comparison with the case in Figure 11, which means that the prediction model $q^{\prime \prime }=0.549144w_{m}^{3}+0.649612w_{m}-2.923254$ gives rise to a larger computational error due to the relatively large contact radius *b* (see Figure 10 and Figure 14). Figure 16 shows the variation of the wind pressure *q* with the ratio *C_0_*/*C* of the measured capacitance, where the prediction model $q^{\prime \prime }=-1506.85114{\left(C_{0}/C\right)}^{3}+6135.03677{\left(C_{0}/C\right)}^{2}-8335.21590C_{0}/C+3775.99807$, which is derived from Equation (54), is used to draw the dashed line.

**Figure 17:**
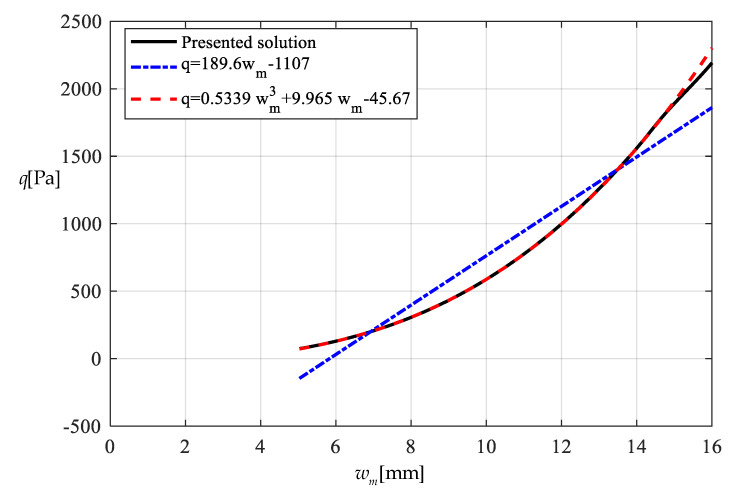
Variations of q with $w_{m}$.

**Figure 18:**
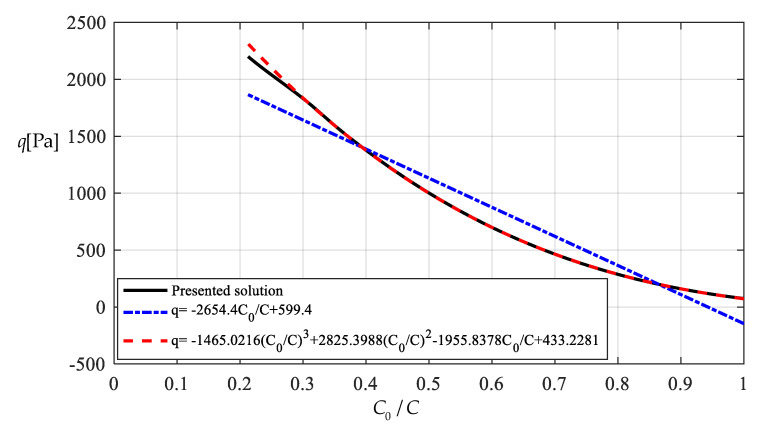
Variations of q with $C_{0}/C$.

It should be noted that as far as the design calibration of sensor is concerned, the numerical calibration here can also be implemented by the method of curve-fitting data, i.e., by using the nonlinear model $q^{\prime \prime }=A_{1}w_{m}^{3}+A_{2}w_{m}+A_{3}$ to fit data in Table 1 or Table 2 with least square method or gradient descent technique, as usually done in the experimental calibration of using a linear model. Table 3 shows the fitting results to the data in Table 2 with least square method, in which the fitted nonlinear model is $q^{\prime \prime }=0.5339\;w_{m}^{3}+9.965\;w_{m}-45.67$, and the fitted linear model is $q^{\prime \prime \prime }=189.6w_{m}-1107$. Figure 17 shows the variations of *q*, $q^{\prime \prime }$ and $q^{\prime \prime \prime }$ with *w_m_*, where the solid line represents the variation of *q* with *w_m_*, the dashed line represents the variation of $q^{\prime \prime }$ with *w_m_*, and the dash-dotted line represents the variation of $q^{\prime \prime \prime }$ with *w_m_*. From Figure 17 and Table 3 it can be seen that the fitting effect of the nonlinear model within 75–2000 Pa has been well improved in comparison with Figure 15. Figure 18 shows the variations of *q*, $q^{\prime \prime }$ and $q^{\prime \prime \prime }$ with the ratio *C_0_*/*C* of the measured capacitance, where the solid line represents the variation of *q* with *C_0_*/*C*, the dash-dotted line is drawn by the linear-fitting model $q^{\prime \prime \prime }=-2654.4C_{0}/C+599.4$, and the dashed line by the nonlinear model $q^{\prime \prime }=-1465.0216{\left(C_{0}/C\right)}^{3}+2825.3988{\left(C_{0}/C\right)}^{2}-1955.8378C_{0}/C+433.2281$. From Table 3 it can be seen that the errors caused by the linear-fitting model has exceeded the 15% allowable error in civil engineering while the errors caused by the nonlinear-fitting model within 75–2000 Pa is about 3%. Therefore, from Table 3 and Figure 17 and Figure 18 it can be concluded that the nonlinear-fitting model $q^{\prime \prime }=A_{1}w_{m}^{3}+A_{2}w_{m}+A_{3}$ is well suitable for the numerical calibration here, while the method of fitting a straight line is unworkable.

## 6. Concluding Remarks

In this paper, an elastic polymer thin film-based capacitive wind-pressure sensor is proposed to meet the anticipated use for real-time monitoring of structural wind pressure in civil engineering. From this study, the following conclusions can be drawn.

The proposed capacitive wind-pressure sensor can eliminate the influence of the change of dielectric constant caused by the change of air humidity. Therefore, it is suitable for use in natural environment.

In comparison with the existing capacitive pressure sensors using soft dielectric elastomer or conductive thin elastic films, the proposed capacitive wind-pressure sensor has a larger pressure-monitoring range, which profits from allowing free choice of polymer films with excellent elasticity. It could implement 100–2000 Pa pressure measurement, and can thus meet the requirements of wind-pressure real-time monitoring in civil engineering.

The closed-form solution presented in this paper has been proved to be basically reliable, and can be used to generate the accurate fitting data for the numerical calibration of the proposed capacitive wind-pressure sensor. The numerical examples conducted show that the numerical calibration here could be conducted directly by the nonlinear-fitting model $q^{\prime \prime }=A_{1}w_{m}^{3}+A_{2}w_{m}+A_{3}$, without having to use the well-known Hencky solution.

However, the present research is only in theoretical stages or provides only a basic theoretical framework for the design of the proposed capacitive wind-pressure sensor, and many details still need to be further studied, especially need to be combined with more comprehensive experimental research.

## Figures and Tables

**Table 1:** The numerical results for *k* = 0.0015 N/mm and Δ*l* = 0.05 mm.

*q* [Pa]	*w_m_* [mm]	*b* [mm]	*q*’’ [Pa]	Relative Errors
68.7	5.0061	0.094574	68.6710	0.042213%
100	5.6598	1.774964	99.3015	0.698500%
150	6.4700	2.163162	148.3866	1.075600%
200	7.1203	2.249843	197.7866	1.106700%
220	7.3496	2.258643	217.5188	1.127818%
230	7.4586	2.260118	227.3463	1.153783%
240	7.5652	2.260116	237.2318	1.153417%
250	7.6694	2.258899	247.1701	1.131960%
300	8.1531	2.240865	296.9366	1.021133%
400	8.9823	2.180269	397.0454	0.738650%
500	9.6827	2.114636	497.3232	0.535360%
800	11.3453	1.946041	799.8860	0.014250%
1000	12.2219	1.858404	999.9076	0.009240%
1500	13.9915	1.695536	1499.9920	0.000533%
2000	15.4000	1.580867	1999.9933	0.000335%
2200	15.8973	1.543461	2199.9967	0.000150%

Relative errors = |*q* −*q"*|/*q*.

**Table 2:** The numerical results for *k* = 0.01 N/mm and △*l* = 0.5 mm.

*q* [Pa]	*w_m_* [mm]	*b* [mm]	*q*’ [Pa]	Relative Errors
75	5.0321	4.6977	70.3192	6.2410%
95	5.4284	5.5773	88.4441	6.9010%
100	5.5170	5.6897	92.8760	7.1240%
150	6.2919	6.1665	137.9497	8.0335%
200	6.9246	6.2119	183.9075	8.0463%
250	7.4657	6.1449	230.4311	7.8276%
300	7.9425	6.0437	277.3756	7.5415%
400	8.7623	5.8240	372.2119	6.9470%
500	9.4592	5.6189	468.0044	6.3991%
600	10.0710	5.4365	564.5354	5.9108%
800	11.1196	5.1321	759.3204	5.0850%
1000	12.0089	4.8889	955.9013	4.4099%
1200	12.7884	4.6889	1153.8950	3.8421%
1400	13.4871	4.5203	1353.0622	3.3527%
1600	14.1233	4.3756	1553.2678	2.9208%
1800	14.7094	4.2490	1754.3664	2.5352%
2000	15.3745	4.1371	2002.7175	0.1359%
2200	16.0270	4.0371	2268.1771	3.0990%

Relative errors = |*q* −*q″*|/*q*.

**Table 3:** The fitting results to the data in Table 2 with least square method.

*q* [Pa]	*w_m_* [mm]	*q*’’’ (Linear-Fitting) [Pa]	Relative Errors	*q*’’ (Nonlinear-Fitting) [Pa]	Relative Errors
75	5.0321	−145.6289	294.1719%	72.5060	3.3253%
95	5.4284	−73.1492	176.9991%	93.8264	1.2354%
100	5.5170	−56.9347	156.9347%	98.9627	1.0373%
150	6.2919	84.7962	43.4692%	150.0177	0.0118%
200	6.9246	200.5032	0.2516%	200.6043	0.3022%
250	7.4657	299.4734	19.7894%	250.8869	0.3548%
300	7.9425	386.6774	28.8925%	300.9783	0.3261%
400	8.7623	536.6332	34.1583%	400.8342	0.2086%
500	9.4592	664.0885	32.8177%	500.4716	0.0943%
600	10.0710	775.9777	29.3296%	600.0327	0.0055%
800	11.1196	967.7824	20.9728%	799.1984	0.1002%
1000	12.0089	1130.4199	13.0420%	998.6217	0.1378%
1200	12.7884	1272.9987	6.0832%	1198.3950	0.1337%
1400	13.4871	1400.7866	0.0562%	1398.5545	0.1032%
1600	14.1233	1517.1518	5.1780%	1599.1406	0.0537%
1800	14.7094	1624.3581	9.7579%	1800.1276	0.0071%
2000	15.3745	1745.9871	12.7006%	2047.7912	2.3896%
2200	16.0270	1865.3335	15.2121%	2311.9721	5.0896%

Relative errors = |*q* −*q″*|/*q* or |*q* −*q‴*|/*q*.

## Appendix A

$$c_{2}=-\frac{1}{16}\frac{1}{\beta^{4}c_{0}^{2}}(24\beta^{3}c_{0}^{2}c_{1}+\tau^{2}),$$

$$c_{3}=\frac{1}{48}\frac{1}{\beta^{5}c_{0}^{3}}(96\beta^{3}c_{0}^{3}c_{1}-4\tau \beta^{2}c_{0}+2\tau^{2}\beta c_{1}+7\tau^{2}c_{0}),$$

$$\begin{array}{l} c_{4}=-\frac{1}{768}\frac{1}{\beta^{8}c_{0}^{5}}(1920\beta^{5}c_{0}^{5}c_{1}+32\beta^{6}c_{0}^{3}-64\tau \beta^{5}c_{0}^{2}c_{1}+24\tau^{2}\beta^{4}c_{0}c_{1}^{2}-160\tau \beta^{4}c_{0}^{3}\\ \;+112\tau^{2}\beta^{3}c_{0}^{2}c_{1}+188\tau^{2}\beta^{2}c_{0}^{3}+\tau^{4}) \end{array},$$

$$\begin{array}{l} c_{5}=\frac{1}{3840}\frac{1}{\beta^{9}c_{0}^{6}}(11520\beta^{5}c_{0}^{6}c_{1}+192\beta^{7}c_{0}^{3}c_{1}-288\tau \beta^{6}c_{0}^{2}c_{1}^{2}+96\tau^{2}\beta^{5}c_{0}c_{1}^{3}+384\beta^{6}c_{0}^{4}\\ \;-1152\tau \beta^{5}c_{0}^{3}c_{1}+576\tau^{2}\beta^{4}c_{0}^{2}c_{1}^{2}-1392\tau \beta^{4}c_{0}^{4}+1272\tau^{2}\beta^{3}c_{0}^{3}c_{1}+1368\tau^{2}\beta^{2}c_{0}^{4}\\ \;-16\tau^{3}\beta^{2}c_{0}+11\tau^{4}\beta c_{1}+22\tau^{4}c_{0}) \end{array},$$

$$\begin{array}{l} c_{6}=-\frac{1}{184320}\frac{1}{\beta^{12}c_{0}^{8}}(9216\beta^{10}c_{0}^{4}c_{1}^{2}-12288\tau \beta^{9}c_{0}^{3}c_{1}^{3}+3840\tau^{2}\beta^{8}c_{0}^{2}c_{1}^{4}+645120\beta^{7}c_{0}^{8}c_{1}\\ \;+32256\beta^{9}c_{0}^{5}c_{1}-66816\tau \beta^{8}c_{0}^{4}c_{1}^{2}+28416\tau^{2}\beta^{7}c_{0}^{3}c_{1}^{3}+31488\beta^{8}c_{0}^{6}-127488\tau \beta^{7}c_{0}^{5}c_{1}\\ \begin{array}{c} \; \end{array}+81216\tau^{2}\beta^{6}c_{0}^{4}c_{1}^{2}-99456\tau \beta^{6}c_{0}^{6}+113472\tau^{2}\beta^{5}c_{0}^{5}c_{1}+88128\tau^{2}\beta^{4}c_{0}^{6}+960\tau^{2}\beta^{6}c_{0}^{3}\\ \;-1920\tau^{3}\beta^{5}c_{0}^{2}c_{1}+816\tau^{4}\beta^{4}c_{0}c_{1}^{2}-3456\tau^{3}\beta^{4}c_{0}^{3}+3000\tau^{4}\beta^{3}c_{0}^{2}c_{1}+2856\tau^{4}\beta^{2}c_{0}^{3}+11\tau^{6}) \end{array},$$

$$\begin{array}{l} c_{7}=\frac{1}{1290240\beta^{13}c_{0}^{9}}(61440\beta^{11}c_{0}^{4}c_{1}^{3}-76800\beta^{10}\tau c_{0}^{3}c_{1}^{4}+23040\beta^{9}\tau^{2}c_{0}^{2}c_{1}^{5}+304128\beta^{10}c_{0}^{5}c_{1}^{2}\\ \;-528384\beta^{9}\tau c_{0}^{4}c_{1}^{3}+203520\beta^{8}\tau^{2}c_{0}^{3}c_{1}^{4}+5160960\beta^{7}c_{0}^{9}c_{1}+511488\beta^{9}c_{0}^{6}c_{1}-1375488\beta^{8}\tau c_{0}^{5}c_{1}^{2}\\ \;+722688\beta^{7}\tau^{2}c_{0}^{4}c_{1}^{3}+324864\beta^{8}c_{0}^{7}-1672704\beta^{7}\tau c_{0}^{6}c_{1}+1315008\beta^{6}\tau^{2}c_{0}^{5}c_{1}^{2}-949248\beta^{6}\tau c_{0}^{7}\\ \;+1310976\beta^{5}\tau^{2}c_{0}^{6}c_{1}-3840\beta^{8}\tau c_{0}^{4}+18624\beta^{7}\tau^{2}c_{0}^{3}c_{1}-22656\beta^{6}\tau^{3}c_{0}^{2}c_{1}^{2}+7632\beta^{5}\tau^{4}c_{0}c_{1}^{3}\\ \;+785664\beta^{4}\tau^{2}c_{0}^{7}+30528\beta^{6}\tau^{2}c_{0}^{4}-77184\beta^{5}\tau^{3}c_{0}^{3}c_{1}+40080\beta^{4}\tau^{4}c_{0}^{2}c_{1}^{2}-66432\beta^{4}\tau^{3}c_{0}^{4}\\ \;+70584\beta^{3}\tau^{4}c_{0}^{3}c_{1}+42744\beta^{2}\tau^{4}c_{0}^{4}-372\beta^{2}\tau^{5}c_{0}+292\beta \tau^{6}c_{0}+507\tau^{6}c_{0}) \end{array},$$

$$\begin{array}{l} c_{8}=-\frac{1}{20643840\beta^{16}c_{0}^{11}}(921600\beta^{14}c_{0}^{5}c_{1}^{4}-1105920\beta^{13}\tau c_{0}^{4}c_{1}^{5}+322560\beta^{12}\tau^{2}c_{0}^{3}c_{1}^{6}\\ \;+5898240\beta^{13}c_{0}^{6}c_{1}^{3}-9216000\beta^{12}\tau c_{0}^{5}c_{1}^{4}+3317760\beta^{11}\tau^{2}c_{0}^{4}c_{1}^{5}+14045184\beta^{12}c_{0}^{7}c_{1}^{2}\\ \;-30523392\beta^{11}\tau c_{0}^{6}c_{1}^{3}+14146560\beta^{10}\tau^{2}c_{0}^{5}c_{1}^{4}+92897280\beta^{9}c_{0}^{11}c_{1}+15151104\beta^{10}c_{0}^{8}c_{1}\\ \;-50817024\beta^{10}\tau c_{0}^{7}c_{1}^{2}+32200704\beta^{9}\tau^{2}c_{0}^{6}c_{1}^{3}+7045632\beta^{10}c_{0}^{9}-44393472\beta^{9}\tau c_{0}^{8}c_{1}\\ \;+42056064\beta^{8}\tau^{2}c_{0}^{7}c_{1}^{2}+15360\beta^{12}c_{0}^{6}-187392\beta^{11}\tau c_{0}^{5}c_{1}+541440\beta^{10}\tau^{2}c_{0}^{4}c_{1}^{2}\\ \;-516096\beta^{9}\tau^{3}c_{0}^{3}c_{1}^{3}+150912\beta^{8}\tau^{4}c_{0}^{2}c_{1}^{4}-19685895\beta^{8}\tau c_{0}^{9}+31984128\beta^{7}\tau^{2}c_{0}^{8}c_{1}\\ \;-282624\beta^{10}\tau c_{0}^{6}+1718784\beta^{9}\tau^{2}c_{0}^{5}c_{1}-2552832\beta^{8}\tau^{3}c_{0}^{4}c_{1}^{2}+1024128\beta^{7}\tau^{4}c_{0}^{3}c_{1}^{3}\\ \;+15432192\beta^{6}\tau^{2}c_{0}^{9}+1352448\beta^{8}\tau^{2}c_{0}^{6}-4179456\beta^{7}\tau^{3}c_{0}^{5}c_{1}+2591424\beta^{6}\tau^{4}c_{0}^{4}c_{1}^{2}\\ \;-2297856\beta^{6}\tau^{3}c_{0}^{6}+2920128\beta^{5}\tau^{4}c_{0}^{5}c_{1}+1268112\beta^{4}\tau^{4}c_{0}^{6}+12096\beta^{6}\tau^{4}c_{0}^{3}\\ \;-24288\beta^{5}\tau^{5}c_{0}^{2}c_{1}+10980\beta^{4}\tau^{6}c_{0}c_{1}^{2}-39648\beta^{4}\tau^{5}c_{0}^{3}+36912\beta^{3}\tau^{6}c_{0}^{2}c_{1}\\ \;+30828\beta^{2}\tau^{6}c_{0}^{3}+73\tau^{8}), \end{array},$$

where β=(1+α)/2, $\tau =(1+2\alpha -3\alpha^{2})/4$.

## Appendix B

$$d_{1}=-\frac{1}{2}\frac{\tau }{\beta c_{0}},$$

$$d_{2}=-\frac{1}{4}\frac{1}{\beta^{2}c_{0}^{2}}(2\beta^{2}c_{0}-\tau \beta c_{1}-\tau c_{0}),$$

$$d_{3}=\frac{1}{96}\frac{1}{\beta^{5}c_{0}^{4}}(32\beta^{5}c_{0}^{2}c_{1}-16\tau \beta^{4}c_{0}c_{1}^{2}+16\beta^{4}c_{0}^{3}-40\tau \beta^{3}c_{0}^{2}c_{1}-16\tau \beta^{2}c_{0}^{3}-\tau^{3}),$$

$$\begin{array}{c} d_{4}=-\frac{1}{192}\frac{1}{\beta^{6}c_{0}^{5}}(48\beta^{6}c_{0}^{2}c_{1}^{2}-24\tau \beta^{5}c_{0}c_{1}^{3}+96\beta^{5}c_{0}^{3}c_{1}-96\tau \beta^{4}c_{0}^{2}c_{1}^{2}+24\beta^{4}c_{0}^{4}\\ -108\tau \beta^{3}c_{0}^{3}c_{1}-24\tau \beta^{2}c_{0}^{4}+5\tau^{2}\beta^{2}c_{0}-4\tau^{3}\beta c_{1}-5\tau^{3}c_{0}) \end{array},$$

$$\begin{array}{l} d_{5}=\frac{1}{1920}\frac{1}{\beta^{9}c_{0}^{7}}(384\beta^{9}c_{0}^{3}c_{1}^{3}-192\tau \beta^{8}c_{0}^{2}c_{1}^{4}+1344\beta^{8}c_{0}^{4}c_{1}^{2}-1056\tau \beta^{7}c_{0}^{3}c_{1}^{3}+1248\beta^{7}c_{0}^{5}c_{1}\\ \;-1968\tau \beta^{6}c_{0}^{4}c_{1}^{2}+192\beta^{6}c_{0}^{6}-1344\tau \beta^{5}c_{0}^{5}c_{1}-192\tau \beta^{4}c_{0}^{6}-40\tau \beta^{6}c_{0}^{3}+112\tau^{2}\beta^{5}c_{0}^{2}c_{1}\\ \;-58\tau^{3}\beta^{4}c_{0}c_{1}^{2}+124\tau^{2}\beta^{4}c_{0}^{3}-152\tau^{3}\beta^{3}c_{0}^{2}c_{1}-87\tau^{3}\beta^{2}c_{0}^{3}-\tau^{5}) \end{array},$$

$$\begin{array}{l} d_{6}=-\frac{1}{23040}\frac{1}{\beta^{10}c_{0}^{8}}(3840\beta^{10}c_{0}^{3}c_{1}^{4}-1920\tau \beta^{9}c_{0}^{2}c_{1}^{5}+19200\beta^{9}c_{0}^{4}c_{1}^{3}-13440\tau \beta^{8}c_{0}^{3}c_{1}^{4}\\ \;+31680\beta^{8}c_{0}^{5}c_{1}^{2}-35040\tau \beta^{7}c_{0}^{4}c_{1}^{3}+18240\beta^{7}c_{0}^{6}c_{1}-40800\tau \beta^{6}c_{0}^{5}c_{1}^{2}+1920\beta^{6}c_{0}^{7}\\ \begin{array}{c} \; \end{array}-19200\tau \beta^{5}c_{0}^{6}c_{1}+160\beta^{8}c_{0}^{4}-1216\tau \beta^{7}c_{0}^{3}c_{1}+2104\tau^{2}\beta^{6}c_{0}^{2}c_{1}^{2}-888\tau^{3}\beta^{5}c_{0}c_{1}^{3}\\ \;-1920\tau \beta^{4}c_{0}^{7}-1232\tau \beta^{6}c_{0}^{4}+5056\tau^{2}\beta^{5}c_{0}^{3}c_{1}-3588\tau^{3}\beta^{4}c_{0}^{2}c_{1}^{2}+2596\tau^{2}\beta^{4}c_{0}^{4}\\ \;-4416\tau^{3}\beta^{3}c_{0}^{3}c_{1}-1554\tau^{3}\beta^{2}c_{0}^{4}+48\tau^{4}\beta^{2}c_{0}-43\tau^{5}\beta c_{1}-56\tau^{5}c_{0}) \end{array},$$

$$\begin{array}{l} d_{7}=\frac{1}{1290240}\frac{1}{\beta^{13}c_{0}^{10}}(184320\beta^{13}c_{0}^{4}c_{1}^{5}-92160\tau \beta^{12}c_{0}^{3}c_{1}^{6}+1198080\beta^{12}c_{0}^{5}c_{1}^{4}\\ \;-783360\tau \beta^{11}c_{0}^{4}c_{1}^{5}+2856960\beta^{11}c_{0}^{6}c_{1}^{3}-2626560\tau \beta^{10}c_{0}^{5}c_{1}^{4}+2972160\beta^{10}c_{0}^{7}c_{1}^{2}\\ \;-4343040\tau \beta^{9}c_{0}^{6}c_{1}^{3}+1198080\beta^{9}c_{0}^{8}c_{1}-3571200\tau \beta^{8}c_{0}^{7}c_{1}^{2}+24576\beta^{11}c_{0}^{5}c_{1}\\ \;-115968\tau \beta^{10}c_{0}^{4}c_{1}^{2}+158976\tau^{2}\beta^{9}c_{0}^{3}c_{1}^{3}-59328\tau^{3}\beta^{8}c_{0}^{2}c_{1}^{4}+92160\beta^{8}c_{0}^{9}\\ \;-1244160\tau \beta^{7}c_{0}^{8}c_{1}+22272\beta^{10}c_{0}^{6}-259584\tau \beta^{9}c_{0}^{5}c_{1}+600384\tau^{2}\beta^{8}c_{0}^{4}c_{1}^{2}\\ \;-325440\tau^{3}\beta^{7}c_{0}^{3}c_{1}^{3}-92160\tau \beta^{6}c_{0}^{9}-122496\tau \beta^{8}c_{0}^{6}+681984\tau^{2}\beta^{7}c_{0}^{5}c_{1}\\ \;-626208\tau^{3}\beta^{6}c_{0}^{4}c_{1}^{2}+217440\tau^{2}\beta^{6}c_{0}^{6}-484608\tau^{3}\beta^{5}c_{0}^{5}c_{1}-118656\tau^{3}\beta^{4}c_{0}^{6}\\ \;-4288\tau^{3}\beta^{6}c_{0}^{3}+10336\tau^{4}\beta^{5}c_{0}^{2}c_{1}-5416\tau^{5}\beta^{4}c_{0}c_{1}^{2}+12608\tau^{4}\beta^{4}c_{0}^{3}\\ \;-14440\tau^{5}\beta^{3}c_{0}^{2}c_{1}-8896\tau^{5}\beta^{2}c_{0}^{3}-43\tau^{7}) \end{array},$$

$$\begin{array}{l} d_{8}=-\frac{1}{5160960}\frac{1}{\beta^{14}c_{0}^{11}}(645120\beta^{14}c_{0}^{4}c_{1}^{6}-322560\beta^{13}\tau c_{0}^{3}c_{1}^{7}+5160960\beta^{13}c_{0}^{5}c_{1}^{5}\\ \;-3225600\tau \beta^{12}c_{0}^{4}c_{1}^{6}+16128000\beta^{12}c_{0}^{6}c_{1}^{4}-13224960\tau \beta^{11}c_{0}^{5}c_{1}^{5}+24514560\beta^{11}c_{0}^{7}c_{1}^{3}\\ \;-28385280\tau \beta^{10}c_{0}^{6}c_{1}^{4}+18144000\beta^{10}c_{0}^{8}c_{1}^{2}-33586560\tau \beta^{9}c_{0}^{7}c_{1}^{3}+177408\beta^{12}c_{0}^{5}c_{1}^{2}\\ \;-665856\tau \beta^{11}c_{0}^{4}c_{1}^{3}+792000\tau^{2}\beta^{10}c_{0}^{3}c_{1}^{4}-271872\tau^{3}\beta^{9}c_{0}^{2}c_{1}^{5}+5483520\beta^{9}c_{0}^{9}c_{1}\\ \;-20885760\tau \beta^{8}c_{0}^{8}c_{1}^{2}+365568\beta^{11}c_{0}^{6}c_{1}-2371584\tau \beta^{10}c_{0}^{5}c_{0}^{2}+4110336\tau^{2}\beta^{9}c_{0}^{4}c_{1}^{3}\\ \;-1888128\tau^{3}\beta^{8}c_{0}^{3}c_{1}^{4}+322560\beta^{8}c_{0}^{10}-5644800\tau \beta^{7}c_{0}^{9}c_{1}+155904\beta^{10}c_{0}^{7}\\ \;-2517504\tau \beta^{9}c_{0}^{6}c_{1}+7421376\tau^{2}\beta^{8}c_{0}^{5}c_{0}^{2}-4976064\tau^{3}\beta^{7}c_{0}^{4}c_{1}^{3}-322560\tau \beta^{6}c_{0}^{10}\\ \;-741120\tau \beta^{8}c_{0}^{7}+5329152\tau^{2}\beta^{7}c_{0}^{6}c_{1}-6096192\tau^{3}\beta^{6}c_{0}^{5}c_{1}^{2}+1191888\tau^{2}\beta^{6}c_{0}^{7}\\ \;-3349056\tau^{3}\beta^{5}c_{0}^{6}c_{1}+14400\tau^{2}\beta^{8}c_{0}^{4}-70848\tau^{3}\beta^{7}c_{0}^{3}c_{1}+98232\tau^{4}\beta^{6}c_{0}^{2}c_{1}^{2}\\ \;-39204\tau^{5}\beta^{5}c_{0}c_{1}^{3}-611712\tau^{3}\beta^{4}c_{0}^{7}-81664\tau^{3}\beta^{6}c_{0}^{4}+248224\tau^{4}\beta^{5}c_{0}^{3}c_{1}\\ \;-159400\tau^{5}\beta^{4}c_{0}^{2}c_{1}^{2}+143840\tau^{4}\beta^{4}c_{0}^{4}-203272\tau^{5}\beta^{3}c_{0}^{3}c_{1}-79216\tau^{5}\beta^{2}c_{0}^{4}\\ \;+947\tau^{6}\beta^{2}c_{0}-892\tau^{7}\beta c_{1}-1182\tau^{7}c_{0}) \end{array},$$

where β=(1+α)/2, $\tau =(1+2\alpha -3\alpha^{2})/4$.
